# Vigilance on New-Onset Atherosclerosis Following SARS-CoV-2 Infection

**DOI:** 10.3389/fmed.2020.629413

**Published:** 2021-01-20

**Authors:** Ya Liu, Hai-Gang Zhang

**Affiliations:** Department of Pharmacology, College of Pharmacy, Army Medical University (Third Military Medical University), Chongqing, China

**Keywords:** COVID-19, SARS-CoV-2, atherosclerosis, endothelial dysfunction, inflammation, angiotensin-converting enzyme 2

## Abstract

The pandemic of coronavirus disease 2019 (COVID-19), caused by SARS-CoV-2, has become a global challenge to public health. While its typical clinical manifestations are respiratory disorders, emerging evidence of cardiovascular complications indicates the adverse interaction between SARS-CoV-2 infection and cardiovascular outcomes. Given that viral infection has emerged as an additional risk factor for atherosclerosis, in this paper, we attempt to clarify the susceptibility to new-onset atherosclerosis in individuals infected with SARS-CoV-2. Mechanistically, serving as functional receptors for SARS-CoV-2, angiotensin-converting enzyme 2 (ACE2) mediates SARS-CoV-2 infection of endothelial cells (ECs) directly, leading to endothelial dysfunction and dysregulation of the renin-angiotensin system (RAS). In addition, high expression of CD147, an alternative receptor, and activation of the NLRP3 inflammasome may also contribute to atherosclerosis in the context of COVID-19. More importantly, SARS-CoV-2 attacks the immune system, which results in excessive inflammation and perpetuates a vicious cycle of deteriorated endothelial dysfunction that further promotes inflammation. The alterations in the blood lipid profile induced by COVID-19 should not be ignored in assessing the predisposition toward atherosclerosis in victims of COVID-19. A better understanding of the underlying mechanisms of SARS-CoV-2 infection and the long-term monitoring of inflammatory factors and endothelial function should be considered in the follow-up of patients who have recovered from COVID-19 for early detection and prevention of atherosclerosis.

## Introduction

Coronavirus disease 2019 (COVID-19) caused by a novel coronavirus, namely severe acute respiratory syndrome coronavirus 2 (SARS-CoV-2), has become a severe public health emergency worldwide. Although its typical clinical manifestations are respiratory dysfunctions, intriguingly, some patients suffering from COVID-19 show cardiovascular symptoms, even as the first symptom (1, 2). Furthermore, patients with prior cardiovascular diseases, such as hypertension and coronary heart disease, tend to have an increased risk of death, highlighting the adverse interaction between SARS-CoV-2 infection and cardiovascular outcomes (3).

Atherosclerosis remains the leading cause of various cardiovascular disorders, including myocardial infarction, stroke, and disabling peripheral artery disease. Although multiple studies have depicted the possible role of viral infection and atherosclerosis since the 1970s (4–6), with less than a year after the outbreak, it certainly appears to still be too early to determine the atherosclerotic risk of COVID-19 victims, which may evolve silently over many years until clinical features occur. However, it is of great concern to note that more than 90% of the confirmed cases will recover because SARS-CoV-2 possesses a lower mortality rate than severe acute respiratory syndrome (SARS) and Middle East respiratory syndrome (MERS), although with more substantial transmission properties (7–9), implying that we are very likely going to face a heavier cardiovascular burden related to atherosclerosis in the future. Consequently, it is necessary to evaluate the risk of atherosclerosis in COVID-19 survivors and to alert people to its complications early. In this paper, we will attempt to clarify the susceptibility to new-onset atherosclerosis in people recovered from COVID-19 as well as pursue the underlying mechanisms.

## Viral Infection and Atherosclerosis

Established risk factors for atherosclerosis, such as hyperlipidemia, hypertension, and smoking, have been efficaciously reduced, however, the occurrence of atherosclerotic disease is still high. In addition, 30–50% of patients actually lack these traditional risk factors, suggesting that other factors are involved in atherosclerotic pathogenesis (10). Clinical data have shown a higher prevalence of subclinical atherosclerosis in human immunodeficiency virus-infected patients (HIV^+^) than in HIV^−^ subjects, independent of the traditional atherosclerosis risks (11, 12). Furthermore, vulnerable plaque characteristics are more common among HIV^+^ patients than among control individuals (13). Clinical observations have indicated that the atherosclerosis risk in patients with hepatitis C is approximately double and the severity is higher (14). A prospective cohort study performed in Japan revealed that human T-cell leukemia virus-1 (HTLAV-1) infection could emerge as an independent predictor of increased carotid intima–media thickness (CIMT) (15). In addition, cytomegalovirus (CMV), Epstein-Barr virus (EBV), influenza viruses, herpes simplex virus-1 (HSV-1), and HSV-2 have also been demonstrated to be closely related to atherogenesis or atherosclerosis-related events in human and animal models (16–18). It is therefore speculated that viral infection has a potential implication in atherosclerosis. Several studies has proposed “direct” mechanisms due to the presence of viral pathogens within atherosclerotic lesions but not within normal blood vessels (19, 20). The virus can enter, lay dormant or replicate in cells and then exert local pro-atherosclerotic effects, including endothelial dysfunction, leukocytes transmigration, vascular smooth muscle cell proliferation, thrombosis and plaque rupture, along with a chronic inflammatory environment in the vessel wall. Regardless of whether viral pathogens are detected *in situ* in the plaque, indirect effects of non-vascular infections leading to systemic inflammation have been related to atherosclerosis. The imbalanced immune response, elevates oxidative stress and disturbs autophagy, which can contribute to the production of plasma inflammatory factors (21, 22). However, mechanistic experimental studies regarding virus-associated atherosclerosis are very limited.

## Direct Influences of SARS-CoV-2 on Atherosclerosis

To better determine the susceptibility to atherosclerosis in COVID-19 survivors, it is vital to learn about SARS-CoV-2 and understand how virus-host interactions manifest as risk factors. Accordingly, the risk factors can delineate regulatory programs that mediate atherosclerotic occurrence, provide valuable clues about disease determinants, and help establish appropriate public health measures.

### SARS-CoV-2, ACE2 and Atherosclerosis

#### ACE2-Mediated Endothelial Dysfunction

**Figure 1:**
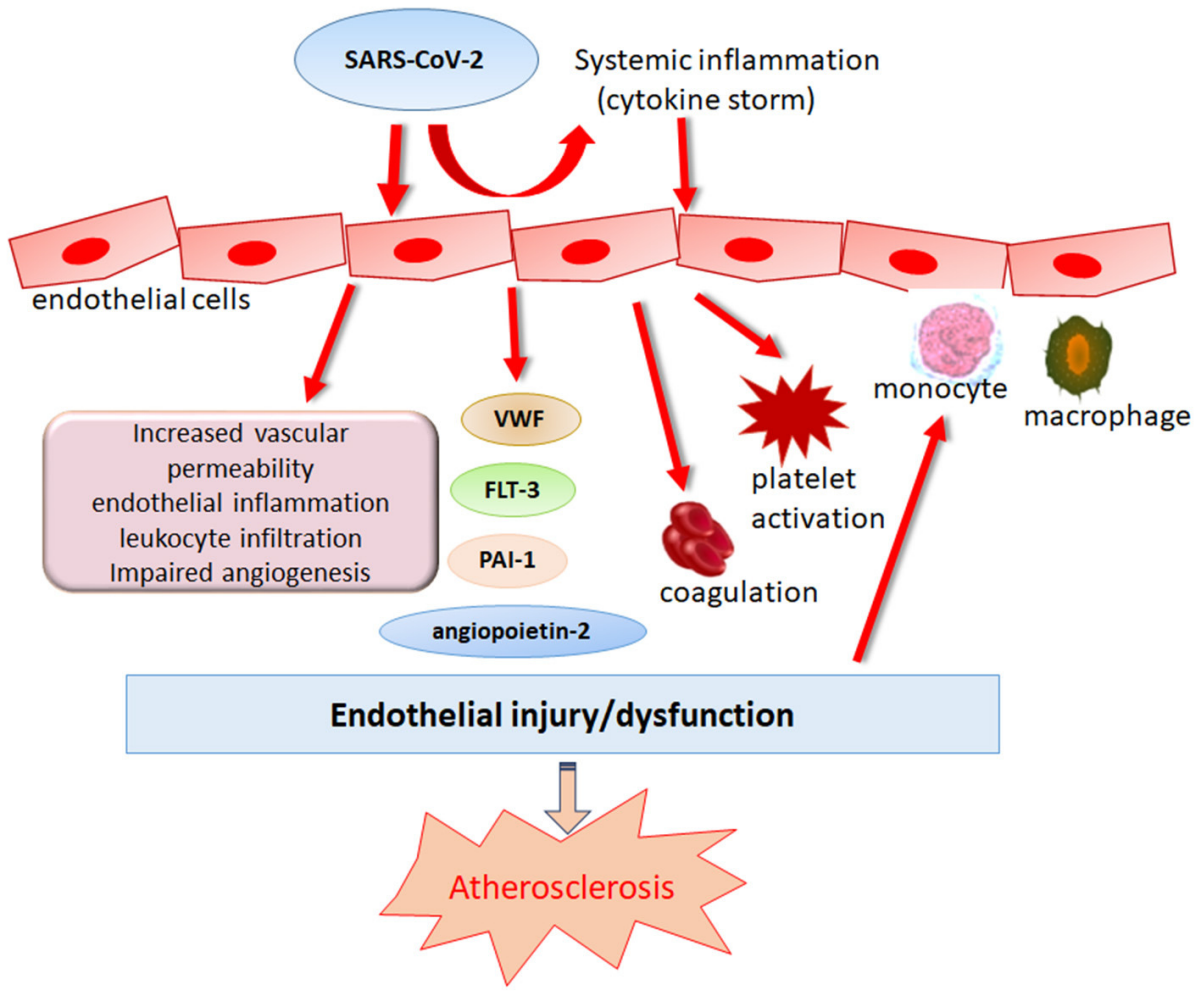
Role of SARS-CoV-2 in endothelial dysregulation. Endothelial dysfunction is an initial step in the development of atherosclerosis that precedes clinical symptoms. SARS-CoV-2 can induce endothelial damage directly or indirectly by eliciting immune dysregulation which causes cytokine storm, leading to the deteriorations of endothelial damage.

Coronaviruses are enveloped viruses, consisting of a set of structural proteins that include spike (S), envelope (E), membrane (M), and nucleocapsid (N) proteins. Among these proteins, the S protein can bind to the membrane receptor on host cells, thus gaining entry into cells and replicating potential in human cells. Similar to SARS-CoV, SARS-CoV-2 also utilizes angiotensin-converting enzyme 2 (ACE2) for cell attachment and infection through the S protein (23). Host transmembrane protease serine 2 (TMPRSS2) cleaves spike protein, which is a necessary step for virus fusion to cellular membranes and entry into the cell (24). SARS-CoV-2 has a higher affinity for binding to ACE2 than SARS-CoV, and binding involves more substantial numbers of interaction sites (25). ACE2 is widely expressed in cardiovascular tissue, including endothelial cells (ECs), in support of a possible mechanism of direct viral injury (26). Notably, circulating endothelial cells are elevated in patients admitted to the hospital with COVID-19 (27). Varga et al. provided microscopic evidence of SARS-CoV-2 viral particles in ECs and diffuse endothelial inflammation (28). *In vitro*, SARS-CoV-2 has been proven to infect engineered human blood vessel organoids directly (29). The plasma levels of Von Willebrand factor (VWF), angiopoietin-2, Fms-related tyrosine kinase 3 ligand (FLT-3L), and plasminogen activator inhibitor type (PAI)-1 are significantly elevated in patients with COVID-19, further supporting the hypothesis of SARS-CoV-2-induced endothelial dysfunction or damage (30, 31). In addition, researchers in Italy and the UK found a significant increase in the incidence of Kawasaki-like disease among children who tested positive for SARS-CoV-2 or were potentially exposed to SARS-CoV-2, highlighting the importance of SARS-CoV-2 infection in coronary artery abnormalities (32, 33). Taken together, these studies point to endothelial SARS-CoV-2 infection as a possible direct trigger of endothelial adverse effects (Figure 1). Endothelial dysfunction is an initial step in the development of atherosclerosis that precedes clinical symptoms and has prognostic value for future cardiovascular events (34, 35). Furthermore, endothelial dysfunction emerges as one of the essential mechanisms corresponding to the enhanced atherosclerotic risk among HIV, HCV and other viral infected people (14, 36, 37). Therefore, endothelial dysfunction induced by SARS-CoV-2 infection indeed becomes a strong contributor to upcoming atherosclerosis in subjects who have recovered from COVID-19.

### Dysregulation of RAS

Well-known as a negative regulator of the renin–angiotensin system (RAS) with the ability to cleave angiotensin-II (Ang-II) into the vasodilator Ang-(1-7), ACE2 has been documented to have pleiotropic beneficial actions in the process of atherosclerosis. Ang-(1-7) appears particularly important in the antiatherosclerotic effects of ACE2. Sahara et al. revealed that the deletion of ACE2 promotes the development of Ang-II-mediated vascular inflammation and atherosclerosis in apolipoprotein E knockout mice (38). Overexpression of ACE2 could reduce atherosclerotic lesion size and increase the collagen content of plaques (39). Alternatively, Ang-(1-7) treatment was also shown to prevent early atherosclerosis and enhance plaque stability (40, 41). However, in the context of SARS-CoV-2 infection, binding via ACE2 results in downregulation of membrane-bound ACE2 and the concurrent loss of catalytic activity of ACE2 in the RAS system, which leads to a decrease in the level of Ang-(1-7) and an increase in Ang-II concentration. In contrast to ACE2/Ang-(1-7), Ang-II can promote proliferation, inflammation and oxidative stress, contributing to atherosclerosis development (42, 43). Thus, SARS-CoV-2 entry is expected to shift the RAS balance from the protective ACE2-Ang-(1-7) arms to the detrimental ACE-Ang-II axis, implying that the inhibition of atherosclerosis from ACE2/Ang-(1-7) is weakened, however, acceleration of atherosclerosis from Ang-II is enforced. Notably, dysregulation of RAS seems to be independent of ongoing SARS-CoV-2 infection.

Accumulating evidence has shown that angiotensin-converting enzyme inhibitors (ACEIs) and angiotensin receptor blockers (ARBs) exert numerous beneficial actions on cardiac and vascular structure and function beyond their blood pressure-lowering effects (44, 45). In principle, the use of ACEIs and ARBs that produce endothelial protective effects could alleviate COVID-19 symptoms and potentially reduce the severity of the disease (46). However, concerns have been raised regarding whether individuals on ACEIs/ARBs are at a greater risk of SARS-CoV-2 infection and COVID-19 exacerbation, as this class of drugs is suspected to be a risk factor for ASRS-CoV-2 infection by upregulating ACE2 (47, 48). Remarkably, a large consecutive cohort study of 1,200 patients in the UK and a multicenter retrospective study in China both support the beneficial effects of RAS inhibitors in patients with COVID-19 and so far, there is no evidence for the potential adverse effect of these agents in patients with COVID-19 (49, 50). However, whether treatment with ACEIs/ARBs can decrease the incidence of atherosclerosis in COVID-19 survivors needs long-term follow-up research.

### SARS-CoV-2, CD147, and Atherosclerosis

A current study elegantly found that CD147 can potentially bind to SARS-CoV-2, providing an additional infection route (51). CD147 is a transmembrane glycoprotein that belongs to the immunoglobulin superfamily and expressed at varying levels in many cell types in its different glycoforms (52). Similar to SARS-CoV-2-induced pulmonary damage, CD147 levels are increased in patients with chronic obstructive pulmonary disease (53). Meplazumab, a humanized anti-CD147 antibody, has been shown to inhibit SARS-CoV-2 replication *in vitro* (51). Recently, an open label, concurrent controlled add-on clinical trial in China revealed that the percentage of improvement in patients with severe COVID-19 presentations seems to be higher in patients receiving weekly treatment with meplazumab than in patients receiving conventional treatment. In addition to viral clearance, meplazumab is likely to facilitate restoration of normal lymphocyte counts and decrease C-reactive protein (CRP) levels (54). SARS-CoV-2 has been found to efficiently infect immune cells expressing low ACE2, such as macrophages and T lymphocytes, through CD147-mediated viral entry (55). Therefore, CD147 is upregulated and possibly participates in hyperinflammation induced by SARS-CoV-2. Accumulating studies have highlighted the potential proatherosclerotic effects of CD147 in atherosclerosis (56). Furthermore, statins achieve antiatherosclerotic roles that partly rely on downregulation of CD147 (57). Of note, statins have been recommended to serve as add-on or coadjuvant therapy against COVID-19 (58), strongly suggesting that SARS-CoV-2 infection and atherosclerosis tend to both experience similar pathological processes related to CD147.

### SARS-CoV-2 and the NLRP3 Inflammasome

Following an RNA viral infection, the host cell response involves the activation of the Nod-like receptor family pyrin domain-containing three (NLRP3) inflammasome, leading to secretion of the proinflammatory cytokines interleukin (IL)-1β and IL-18 (59). Accumulating evidence has indicated that NLRP3 recognizes RNA viruses by sensing the cellular distress induced by viroporins (60–62). Viroporins are small virus-encoded proteins that are able to permeabilize membranes for ions by forming membrane channels (63, 64). It has been shown that the E protein of SARS-CoV can form Ca^2+^ permeable ion channels, thereby activating the NLRP3 inflammasome (63). SARS-CoV-2 shares many biological features with SARS-CoV owing to the 79.6% genomic sequence identity (65), which implies that SARS-CoV-2 also has the ability to activate the NLRP3 inflammasome. A subsequent study found another viroporin in SARS-CoV, namely 3a protein, which is responsible for activation of the NLRP3 inflammasome (66). The 3a protein is also present in the SARS-CoV-2 genome, raising the possibility that SARS-CoV-2 enables direct activation of the NLRP3 inflammasome (67). In COVID-19, dysregulation of the NLRP3 inflammasome in monocytes and macrophages seems to be involved in a hyperinflammatory state contributing to severe tissue damage (68, 69). The first clinical study of an NLRP3 inflammasome inhibitor (tranilast) to treat COVID-19 is ongoing in China. The activated NLRP3 inflammasome has been widely linked to a large number of diseases, and several experimental studies highlighted that atherosclerosis may not be intrinsically caused by the NLRP3 inflammasome, but is closely linked to and often aggravated by NLRP3 inflammasome activation (70). Thus, the NLRP3 inflammasome probably fuels inflammation in the context of COVID-19 to promote the progression of atherosclerosis.

## COVID-19 and Systemic Inflammation

**Figure 2:**
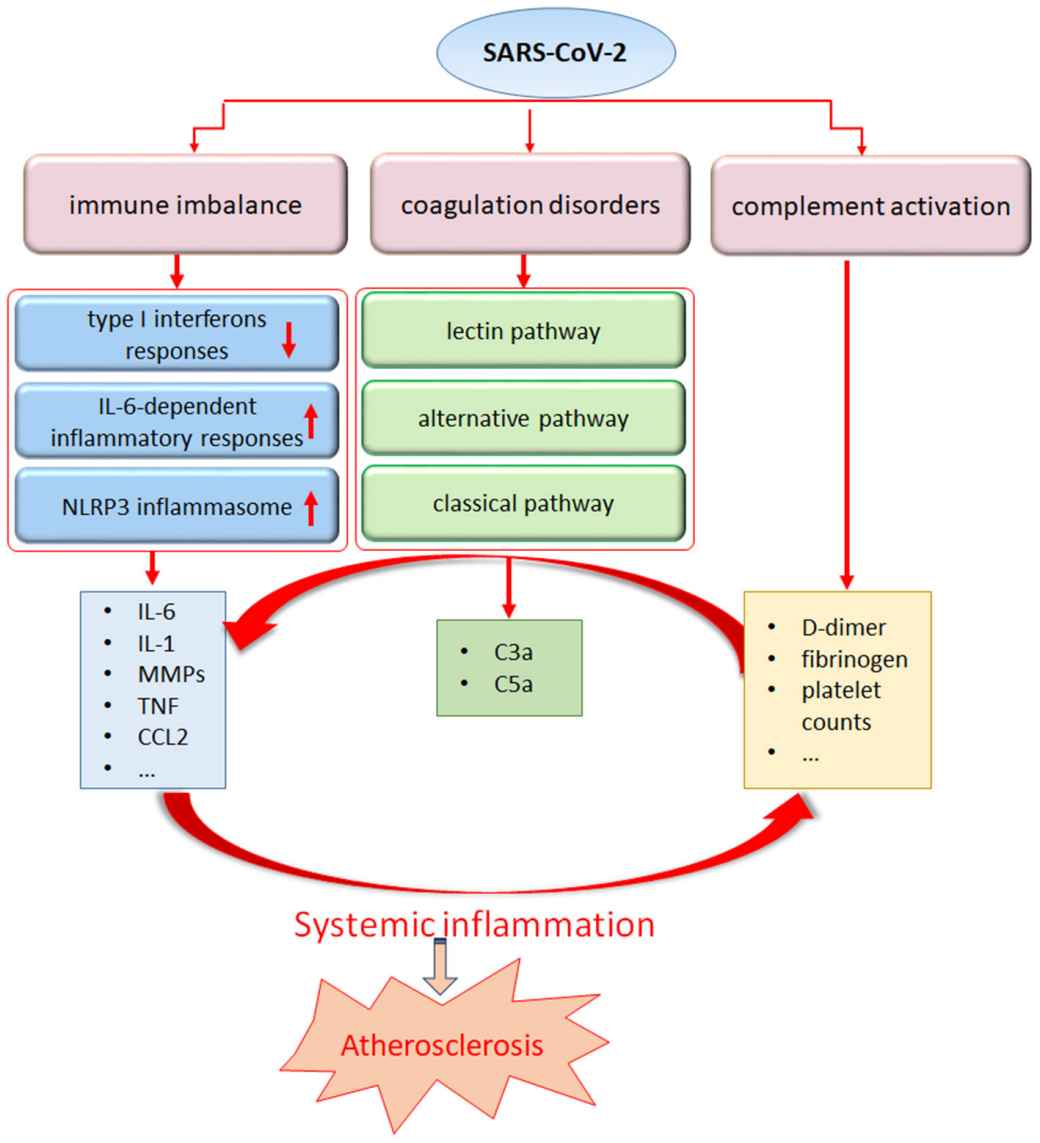
The systemic inflammatory response from SARS-Cov-2 may trigger atherosclerosis. SARS-CoV-2 infection can stimulate a pronounced immune response including dysregulation of interferon and IL-6 pathway, NLRP3 inflammasome activation, leading to an uncontrolled production of proinflammatory mediators. In addition, coagulation disorders and complement abnormalities induced by SARS-CoV-2 infection may contribute to the maladaptive inflammatory response.

In addition to the lungs, immune organs are the second most attacked system by SARS-CoV-2. An excessive inflammatory response to SARS-CoV-2, referred to as a cytokine storm, has been implicated in COVID-19 severity and death, as evidenced by the increased levels of CRP, IL-6, IL-7, tumor necrosis factor (TNF) and inflammatory chemokines, including CC- chemokine ligand 2 (CCL2), CCL3 and CXC- chemokine ligand 10 (CXCL10), as well as IL-2 receptor. Higher levels of IL-6 in the serum have been linked to a worse prognosis in patients suffering from SARS-CoV-2 infection (1, 3). Accompanied by the uncontrolled cytokine response, the presence of a global T cell lymphopenia serves as a common feature in patients with COVID-19 and is particularly prominent in severe patients (71). Furthermore, the T cell numbers appear to be negative correlated with the serum levels of TNF, IL-6 and IL-10 (72). In addition to immune factors such as type I interferons and dysregulation of IL-6-dependent inflammatory responses (73), several retrospective observational studies of patients have shown that SARS-CoV-2 engages robust activation of complement and coagulation cascades (74–76). Elevated levels of D-dimer and fibrinogen, with minor abnormalities in prothrombin time, activated partial thromboplastin time, and increased platelet counts, have been detected in the initial stage of SARS-CoV-2 infection (77). A case series from New York reported large-vessel ischemic stroke in five patients infected with SARS-CoV-2 (78). Furthermore, acute limb ischemia was also described in 20 infected patients in a case series from Italy. All 20 patients were diagnosed with COVID-19- related pneumonia before acute limb ischemia was detected (79). At present, anticoagulation treatment has been linked to survival in patients hospitalized with COVID-19, and a wide range of clinical trials are evaluating the use of low-molecular-weight heparin to treat patients with COVID-19 (80–82). While encountering systemic inflammation, exposure of the endothelium to an array of proinflammatory cytokines may also act as a key source of inflammatory cytokines, leading to aggravated endothelial damage and amplified vascular and systemic inflammation accompanied by an imbalance of pro- and anticoagulant pathways (Figure 2).

It is highly acknowledged that atherosclerosis is, in fact, an inflammatory process with innate and adaptive immune activation that plays a part in the entire disease. Our previous studies have suggested that systemic inflammation induced by zymosan could accelerate the progress of atherosclerosis in high fat diet-treated rabbits and rats, and the imbalance of the cytokine network was responsible for deteriorated lipid disorders and advanced atherosclerotic plaques (83, 84). Indeed, diseases with a proinflammatory state, such as rheumatoid arthritis and systemic lupus erythematosus, entail an elevated risk of atherosclerosis (85, 86). Canakinumab, a fully humanized monoclonal antibody that targets IL-1β, has received approval in many countries as an orphan drug to treat rare heritable chronic inflammatory diseases (87). Currently, several clinical trials concerning canakinumab are in progress to test the inflammatory hypothesis of atherosclerosis (88). Of note, clinical trials to assess cytokine blockade, including canakinumab and tocilizumab (targeting IL-6) in patients with COVID-19 are ongoing. Accordingly, inflammation engendered by SARS-CoV-2 infection represents one such state that shares the common pathophysiological milieu of atherosclerosis.

## Dyslipidemia and COVID-19

To date, the study of the blood lipid profile related to COVID-19 is in its infancy. Two retrospective studies were performed to underline a sharp decrease in high-density lipoprotein (HDL) levels in patients with severe COVID-19. However, there is no consensus regarding the value of serum total cholesterol (TC), low-density lipoproteins (LDL) and triglyceride (TG) (89, 90). A 12-year follow-up study based on 25 SARS survivors demonstrated that 68% of victims had significant alterations in lipid metabolism, which correlated with hyperlipidemia, cardiovascular abnormalities, and abnormal glucose metabolism (91). Altered serum lipid concentrations have been documented to appear during viral infection, including HIV and HCV (12, 14, 92). In addition, high-dose pulses of methylprednisolone, antiviral drugs including liponovir/ritonavir, and tocilizumab have all been reported to be associated with disturbed lipid metabolism (93–95). Multiple lines of incontrovertible evidence have proven a causal role for high-serum LDL in atherosclerosis. In general, LDL activates intracellular pathways to increase local and systemic inflammation, monocyte adhesion, endothelial cell dysfunction and apoptosis, and smooth muscle cell proliferation, resulting in foam cell formation and the genesis of atherosclerotic plaques. In contrast, HDL is capable of preventing or attenuating atherosclerosis (96, 97). Although the blood lipid profile requires long-term monitoring, the direct participation of hyperlipidemia should not be discarded in assessing the risk of atherosclerosis in COVID-19 survivors.

## Conclusion

Accumulating evidence has indicated that EC dysfunction is a central feature of COVID-19, accordingly, the major link to SARS-CoV-2-induced atherosclerosis may be centered on endothelial cells. It is proposed that the endothelial dysfunction and injury occurring in COVID-19 reflects direct infection of ECs by SARS-CoV-2 in receptor-dependent and independent manners. The indirect bystander injury resulting from systemic inflammation further amplifies endothelial dysfunction, perpetuating a vicious cycle of endothelial dysfunction that promotes inflammation. It has been appreciated that there is not a specific virus or pathogen that initiates atherosclerosis but rather the inflammatory level and its chronicity and intensity. To date, our knowledge and understanding of COVID-19-associated atherosclerosis is limited by what is known about traditional atherosclerosis because current knowledge has been gained almost exclusively through clinical studies. There is a pressing need to experimentally unravel the missing link between SARS-CoV-2 and atherosclerosis.

## Author Contributions

YL wrote and drafted of the article. H-GZ revised the manuscript critically. All authors contributed to the article and approved the submitted version.

## Conflict of Interest

The authors declare that the research was conducted in the absence of any commercial or financial relationships that could be construed as a potential conflict of interest.
